# The Effect of a Diet Enriched with *Jerusalem artichoke*, Inulin, and Fluoxetine on Cognitive Functions, Neurogenesis, and the Composition of the Intestinal Microbiota in Mice

**DOI:** 10.3390/cimb45030168

**Published:** 2023-03-21

**Authors:** Aleksandra Szewczyk, Marta Andres-Mach, Mirosław Zagaja, Agnieszka Kaczmarczyk-Ziemba, Maciej Maj, Joanna Szala-Rycaj

**Affiliations:** 1 Department of Experimental Pharmacology, Institute of Rural Health, Jaczewskiego 2, 20-090 Lublin, Poland; 2 Department of Evolutionary Genetics and Biosystematics, Faculty of Biology, University of Gdansk, Wita Stwosza 59, 80-308 Gdansk, Poland; 3 Department of Biopharmacy, Medical University of Lublin, Chodzki 4A, 20-093 Lublin, Poland

**Keywords:** topinambur, inulin, fluoxetine, prebiotics, intestinal microbiota, neurogenesis, cognitive functions

## Abstract

The aim of the study was to assess the effect of long-term administration of natural prebiotics: *Jerusalem artichoke* (topinambur, TPB) and inulin (INU) as well as one of the most popular antidepressants, fluoxetine (FLU), on the proliferation of neural stem cells, learning and memory functions, and the composition of the intestinal microbiota in mice. Cognitive functions were assessed using the Morris Water Maze (MWM)Test. Cells were counted using a confocal microscope and ImageJ software. We performed 16S rRNA sequencing to assess changes in the gut microbiome of the mice. The obtained results showed that the 10-week supplementation with TPB (250 mg/kg) and INU (66 mg/kg) stimulates the growth of probiotic bacteria, does not affect the learning and memory process, and does not disturb the proliferation of neural stem cells in the tested animals. Based on this data, we can assume that both TPB and INU seem to be safe for the proper course of neurogenesis. However, 2-week administration of FLU confirmed an inhibitory impact on Lactobacillus growth and negatively affected behavioral function and neurogenesis in healthy animals. The above studies suggest that the natural prebiotics TPB and INU, as natural supplements, may have the potential to enrich the diversity of intestinal microbiota, which may be beneficial for the BGM axis, cognitive functions, and neurogenesis.

## 1. Introduction

According to the most recent scientific estimates, there are about 3.8 × 10^13^ bacteria in the adult human body, which is 1.3 times more than human cells [1]. The totality of microorganisms in a specific environment is called the microbiota, and the collective genomes of all microorganisms are called the microbiome [2]. The human microbiome includes not only bacteria but also other microorganisms such as fungi, archaea, viruses, and protozoa [3].

The composition of the intestinal microbiota is an outcome of an interplay between numerous variables, such as a diet, an environment, host genetics, an exposure to infections, and an antibiotic usage [4]. Most of the human intestinal bacteria belong to four phyla: *Firmicutes*, *Bacteroidetes*, *Actinobacteria*, and *Proteobacteria*, with the *Firmicutes* and *Bacteroidetes* predominating. The equilibrium of the gut’s diverse microbial population is essential for preserving the host’s state of health [5].

In general, gut microbiome is beneficial for the human host. These microorganisms are key players in regulating gut metabolism, and are necessary to understand metabolism dysfunctions. For instance, there are connections between gut bacteria and the synthesis of vitamins B and K, short-chain fatty acid (SCFA) production, pathogen growth inhibition, preservation of intestinal barrier integrity and mucosal immune homeostasis, and involvement in the xenobiotic metabolism system [6,7].

The gut, the gut microbiota, and the brain are involved in a constant two-way communication that is referred to as the brain–gut–microbiome (BGM) axis [8,9]. Research on the BGM axis primarily includes the studies of pre- or probiotics as well as antibiotics in animal models [10,11,12,13], studies in germ-free (GF) animals [14], and fecal transplants [15]. This allows the identification of pathways that regulate BGM signaling between the digestive tract and the brain, including neural, endocrine, and immune pathways. It has been shown that the BGM axis plays an important role in the formation and maintenance of cognitive functions [16,17,18,19]. Moreover, the BGM axis was proved to be significant in the regulation of neurogenesis, one of the most essential processes of proliferation, migration, and differentiation of new neural cells [20,21,22,23]. Undoubtedly, one of the key factors negatively affecting the BGM and the neurogenesis is stress [24,25,26,27].

BGM is crucial in maintaining a proper homeostasis, and several psychiatric and nonpsychiatric illnesses have been proved to be at least in part responsible its dysfunction [28,29,30]. It should also be noted that, in addition to diseases, the composition and diversity of the gut microbiota can be affected by drugs used to treat the disorder, especially antibiotics. However, recent studies have shown that non-antibiotic drugs, such as the antipsychotics and antidepressants, also affect the intestinal microbiota [31,32]. Few studieshave been done so far to determine how the antidepressants affect the microbiota in the gut. One of them concerns research on fluoxetine hydrochloride (FLU), an antidepressant drug belonging to the group of selective serotonin reuptake inhibitors (SSRIs) used in depression, panic attacks, anxiety, or obsessive–compulsive symptoms [33,34]. The in vitro studies carried out so far have provided information that FLU has antimicrobial activity [35], whereas in vivo studies have shown its negative effect on the composition of the intestinal microbiome in both rats [35] and mice [36].

For years, we utilized probiotic bacteria in addition to food to maintain a healthy microbiome or to rebalance the system [37]. Probiotics are live bacteria and yeasts that have a beneficial effect onhuman health. The impact of probiotics may also be favored by prebiotics, which can be used as an alternative to probiotics or as their additional support [38]. Prebiotics are non-digestible food components that specifically promote the development of probiotic bacteria in the gut, including lactobacilli and bifidobacteria [39]. However, various prebiotics will promote the growth of different native gut bacteria [38]. The presence of prebiotics in the diet hasmany beneficial effects on the gut, the immune system, and brain function, particularly, brain-derived neurotrophic factor (BDNF) expression and N-methyl-D-aspartate (NMDA) receptor signaling. Substances classified as prebiotics are oligosaccharides, including galactooligosaccharides(GOS), transgalactooligosaccharides(TOS), xylooligosaccharides(XOS), fructooligosaccharides(FOS), isomaltooligosaccharides(IMO), as well as polysaccharides such as inulin (INU), cellulose, pectin, hemicellulose, or reflux starch [38].

INU is composed of fructose residues connected by β-(2,1) glycosidic linkages, and it plays the role of spare material in plants. INU is abundantly present in a variety of root vegetables including topinambur(TPB), banana, chicory, leek, and onion. TPB, a tuberous perennial plant of the *Asteraceae* family commonly known as wild sunflower or *Jerusalem artichoke*, has 160–200 g of INU per kilogram of fresh weight [40,41]. According to the study done thus far, TPB has several health-promoting qualities, including lowering blood glucose, triglycerides, LDL cholesterol, and total cholesterol [42]. In addition, TPB supplementary diet has an additive impact on the probiotic bacteria in the gut of rats [43] and mice [41].

Taking into account the above data, the aim of this study was to determine the impact of the long-term supplementation with prebiotics, TPB, and INU, in the form of natural compounds and 2-week administration of the antidepressant drug FLU, on the development of probiotic bacteria necessary for the proper functioning of the BGM axis, and thus their influence on cognitive functions and neurogenesis in healthy mice.

## 2. Materials and Methods

### 2.1. Animals and Experimental Conditions

All tests were carried out on 6-week-old male C57BL/6J mice (20–25 g). Animals were housed under standard laboratory conditions (natural light–dark cycle, 55 ± 5% humidity, and a temperature of 21 ± 1 °C) and allowed food (a complete feed for mice and rats; AGROPOL, Marynin, Poland) and water ad libitum. After a 7-day acclimatization period the animals were randomly assigned to four experimental groups (TPB, INU, FLU, and control) consisting of seven mice each. All experimental procedures were approved by the Local Ethics Committee at the University of Life Science in Lublin (No 73/2020).

### 2.2. Drugs

The following substances and drugs were used in the presented study: TPB (Organic, Sieniawa, Poland), fluoxetine hydrochloride (FLU; Sigma Aldrich, St. Louis, MO, USA), INU isolated from chicory roots (FORMEDS, Poznań, Poland), 5-bromo-2′-deoxyuridine BrdU(Sigma Aldrich, St. Louis, MO, USA), medetomidine hydrochloride (Tocris Bioscience, Bristol, UK), isoflurane (Baxter, Warszawa, Poland), methylscopolamine(Sigma Aldrich, St. Louis, MO, USA). All substances were suspended in water for injections (Baxter, Poland). TPB, INU, and FLU administrated via gastric gavages, whereas BrdU and medetomidine hydrochloride were administrated via intraperitoneal (i.p.) injections. All substances were administrated with 1 mL syringes in a volume of 10 mL/kg.

### 2.3. Drugs Administration

Animals were divided into fourgroups (sevenmice per group, *n* = 7):

1. TPB
2. INU
3. FLU
4. control group (water for injection)

**Figure 1:**
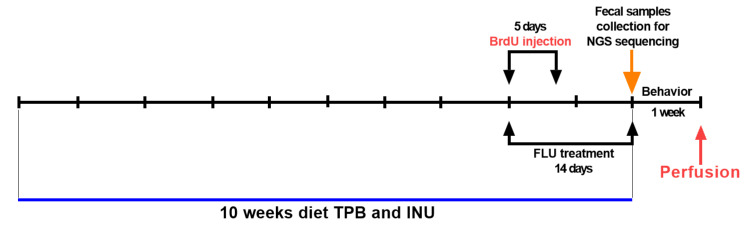
Schematic illustration of the experimental design.

The supplementation lasted for 10 weeks (Figure 1). The animals were given a freshly prepared suspension of powdered TPB, INU, and FLU via an oral administration. According to the manufacturer’s recommendation, daily dose forhuman intake of the TPB is 10–15 g per day and INU is 4 g per day. The substances were calculated into the mouse body weight, dissolved in water for injections, and administered once a day (assumed average adult weight of 60 kg, the target dose per kg of body weight was 250 mg-TBP and 66 mg-INU). FLU (12 mg/kg) was administered to the FLU group in the last two weeks of the experiments. The dose of FLU was selected based on the latest literature data [44]. Control animals were given water for injection orally, throughout the duration of the diet. To measure changes in body weight following the 10-week diet, all animals were weighed at the 1st, 3rd, 5th, 7th, and 10th weeks. Additionally, on the 9th week of the diet, the animals were given an i.p. injection of BrdU(a cell proliferation marker) once daily at a concentration of 50 mg/kg for 5 days. Fecal samples were taken to analyze the microbiome after the diet, and the animals underwent the behavioral Morris Water Maze Test (MWM), which measures spatial learning and memory abilities. After the behavioral studies, animals were perfused and their brains were extracted for quantitative analysis of neurogenesis.

### 2.4. Morris Water Maze (MWM) Test

Animals were subjected to behavioral studies10 weeks after the TPB, INU, and FLU diet.

Animals underwent a behavioral MWM test 24 h after the last administration of TPB, INU, FLU, and water for injections (for the control group), according to the methods described earlier [45,46,47]. The MWM is the one of the most commonly used behavioral tests to assess learning and memory processes. In brief, to perform the MWM test, a mouse placed in a circular tank filled with water had to find a platform located above or just below the surface of the water, where it could safely rest. During 5 days of the training test recorded with a TSE (one daily session consisting of four 60-s trials), the animals learned and memorized how to find the platform by means of special signs placed on the walls of the room. On 6th day, the final test was performed. The course of the video tracking system VideoMot2 (TSE Systems, Berlin, Germany) and three parameters were measured: escape latency (the average time needed to find the platform), distance (the average distance traveled in order to find the platform), and time spent in the W-channel.

### 2.5. Fecal Collection, DNA Extraction, and NGS Sequencing

Fresh fecal samples from fivemice in each group (*n* = 5) were collected into sterile Eppendorf tubes and frozenat −80 °C until needed for DNA extraction.

Genomic DNA samples were extracted from about 15 mg of fecal samples using the GeneMATRIX Stool DNA Purification Kit (EurX, Gdańsk, Poland) and following the manufacturer’s protocol. Prior the extraction, all samples were homogenized, and the homogenization step was performed by MP FastPrep-24 Classic instrument (MP Biomedicals, USA) for 1 min in 6.5 m/s. DNA concentrations and the A_260_/A_280_ absorption ratios were assessed using an Eppendorf BioPhotometer D30 spectrophotometer (Eppendorf, Hamburg, Germany). After extraction, the DNA was stored at −20 °C until further use.

The genetic material extracted for 19samples was sent to the Genoplast Laboratory (Poland) for library preparation and 16S sequencing using Illumina MiSeq platform. The V3-V4 hypervariable regions of the bacterial 16S rRNA gene were amplified using primers 341F/785R [48]. Details were congruent with methodology described in Kaczmarczyk-Ziemba et al. [49]. Raw NGS data are deposited and fully available in the Sequence Read Archive (accession number PRJNA912402).

Demultiplexed paired-end reads were imported into QIIME2 (2019.1 release) [50]. The DADA2 algorithm was applied to filter out noise and correct errors in marginal sequences, remove chimeric sequences, merge paired-end reads, and summarize amplicon sequence variants (ASVs) [51]. Taxonomy assignment was performed with a pre-trained SILVA 132 99% OTUs based Naïve-Bayes classifier [52]. ASVs matching with chloroplast and mitochondrial sequences were removed from the dataset for downstream analyses.

#### Biographical Analysis of Microorganisms

Alpha diversity reflects the variation in microbial composition within a single sample. The following indices were used to estimate alpha diversity: Chao1 and Shannon. The Chao1 index (the so-called wealth index) refers to the abundance of individual samples, whereas the Shannon index summarizes the diversity of the population. Analyses were performed for four groups of samples (control, FLU, INU, and TPB). Alpha diversity measures (Chao1 richness index and Shannon diversity index) were calculated using the MicrobiomeAnalyst platform [53,54]. The non-parametric Kruskal–Wallis test was used to compare differences in alpha diversity between different groups.

A fundamental property of microbiomes is beta diversity. It is a measure of the similarity or dissimilarity of samples and quantifies differences in overall taxonomic composition.

### 2.6. Transcardial Perfusion and Brain Slice Preparation

Threeweeks after the last injection of BrdU, the animals were transcardiallyprefused. Immediately prior to perfusion, the mice were i.p. injected with the analgesic medetomidine at a dose of 0.25 mg/kg to relieve pain during surgery. Subsequently, each animal was anesthetized with 2% isoflurane. After opening the mouse’s chest, a cannula was put into theleft ventricle of the heart. Next, saline and 4% paraformaldehyde were injected under equal pressure to fix the brain tissues needed for further research. After perfusion, brains were dissected and cut into 50 µm sections with a Leica vibratome and stored at 4 °C in the cryoprotectant solution. Then, the sections prepared in this way were used for BrdU/NeuN/GFAP staining to determine the effects of TPB, INU, and FLU on the neurogenesis process.

### 2.7. Immunohistochemical Staining-Neurogenesis

For determining the influence of TPB, INU, and FLU on the process of neurogenesis, 50 μm free-floating sections (stored at 4 °C) were immunohistochemically stained to visualize BrdU/NeuN and BrdU/GFAP positive cells according to the methods described previously [45,46,47,55,56].

### 2.8. Confocal Microscopy and Cell Counting

**Figure 2:**
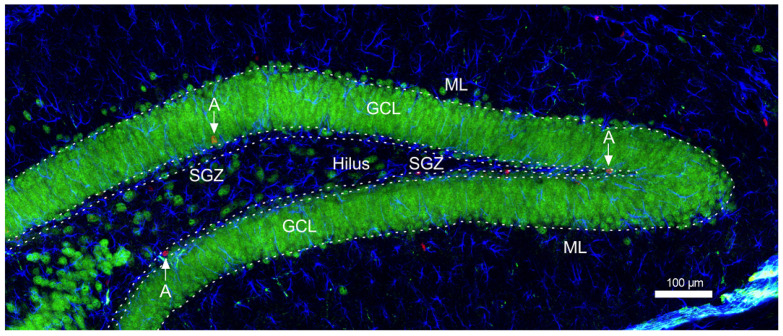
Scheme of areas of the hippocampal DG designated for quantitative analysis of neurogenesis. A—BrdU positive cells; ML—molecular layer; GCL—granule cell layer; SGZ—subgranular zone.

To further establish the phenotype of BrdU labeled cells, we performed a double/triple confocal immunofluorescence staining with BrdU, neuronal nuclei (NeuN), and glial acid fibrillar protein (GFAP) in the granular cell layer (GCL) and subgranular zone (SGZ) of dentate gyrus (DG) using the methods described earlier [45,46,47,55,56] five mice in each group (*n* = 5).The exact area of DG of the hippocampus designated for the quantitative analysis of neurogenesis is presented in Figure 2. Confocal imaging was performed using a Nikon A1R confocal microscope (Tokyo, Japan).

### 2.9. Statistical Analysis of the Results

#### 2.9.1. Statistical Analysis of MWM and Neurogenesis Results

One-way ANOVA was used to analyze the data using the Windows version of the commercial program GraphPad Prism 8.0. (GraphPad Software, San Diego, CA, USA). Bonferroni’s test was then applied for multiple comparisons. Every piece of data is expressed as a mean with standard errors.

#### 2.9.2. Principal Coordinate Analysis (PCoA)

PCoA was used to show species diversity between samples. Primer 7 [57] software was used to calculate the two-dimensional PCoA analysis and to generate the PCoA graph.

#### 2.9.3. ANOSIM and PERMANOVA Analysis

Analysis of similarities (ANOSIM) statistically determines whether the difference between the groups was greater than the difference within the groups. Primer7 software [57] was used to calculate it. In addition, it was used to calculate permutation-based multivariate analysis of variance (PERMANOVA) and to determine if there are significant differences between predefined sample groups.

#### 2.9.4. SIMPER Analysis

The SIMPER analysis was conducted to calculate the overall mean differences between the profiles of the compared groups and to identify the types of bacteria that primarily shape the differences between the microbiomes of the study groups. Past 4.0 [58] was used to perform the SIMPER analysis and pairwise calculation of the overall average differences between the profiles, as well as to identify the bacterial taxa associated primarily with the observed differences between the studied microbiomes.

#### 2.9.5. LEfSe Analysis

To identify bacterial taxa responsible for differences in beta diversity, LEfSe(linear discriminant analysis [LDA] effect size) analysis was performed [59]. The analysis was performed using the MicrobiomeAnalyst platform. Relative taxonomic abundances were used as input to the LEfSe pipeline. The metabolic potential of the gut microbiota was predicted by the Phylogenetic Investigation of Communities by Reconstruction of Unobserved States (PICRUSt2) analysis using the KEGG Pathway database [60].

## 3. Results

### 3.1. Effect of TPB, INU, and FLU Administration on the Body Weight of Healthy Mice

**Figure 3:**
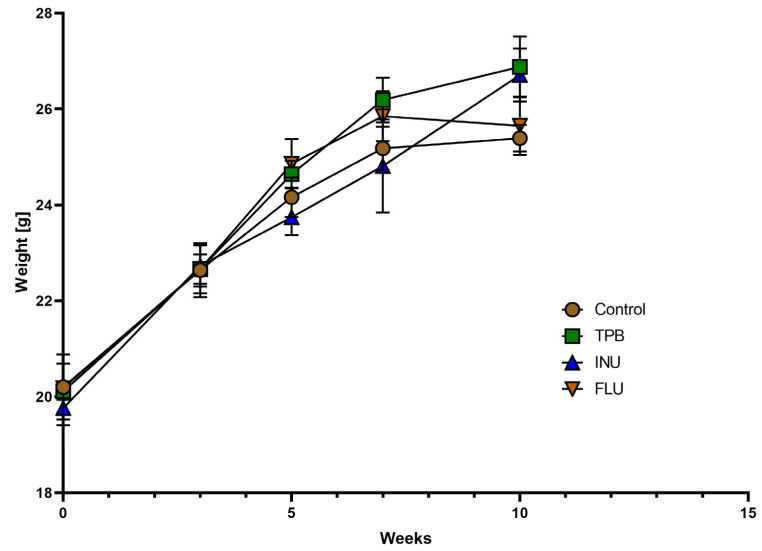
Effect of TPB, INU, and FLU on the body weight of mice. TPB—topinambur; INU—inulin; FLU—fluoxetine. Each bar represents the mean for seven mice; error bars are S.E.M.

Body weight gain analysis of mice receivingTPB, INU, and FLU at the 1st, 3rd, 5th, 7th, and 10th week of the diet showed no significant changes for TPB and INU throughout the duration of the experiments. Two weeks of FLU administration in the 9th and 10th week of the experiment indicated a slight decrease in weight for the FLU mice (Figure 3).

### 3.2. Effect of TPB, INU, and FLU Administration on Mouse Spatial Learning and Memory

In order to assess potential memory and learning disorders as a result of TPB, INU, and FLU administration, the animals were subjected to the Morris Water Maze Test. Three parameters were analyzed: (1) the average time needed to find the platform, (2) the average distance traveled in order to find the platform, and (3) the mean percentageof time spent in the W-channel.

**Figure 4:**
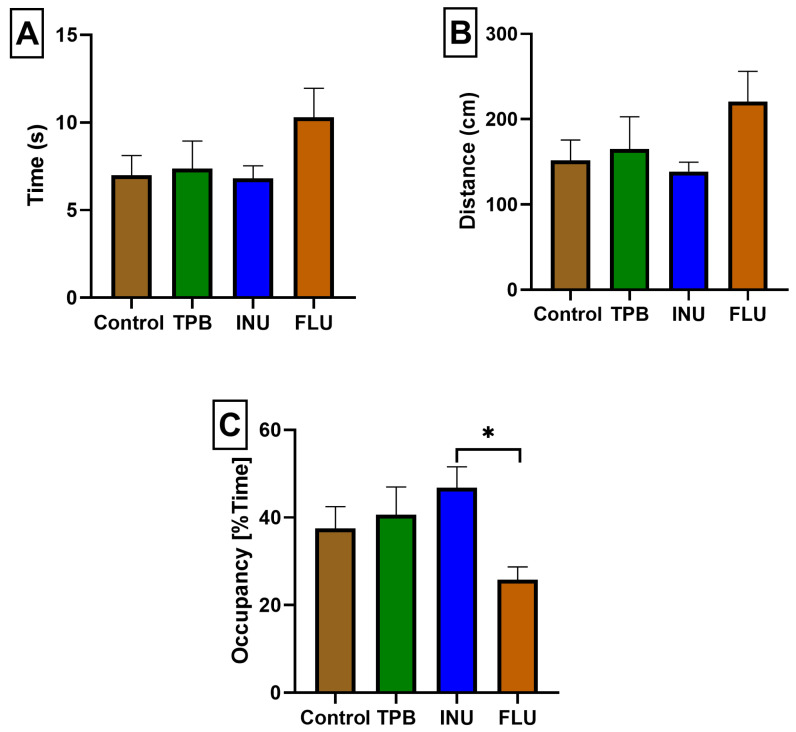
The impact of TPB, INU, and FLU on escape latency (**A**), distance (**B**), and the total average percentage of time spent in the W-channel (**C**). TPB—topinambur; INU—inulin; FLU—fluoxetine. Each bar represents the mean for seven mice; error bars are S.E.M. (* *p* < 0.05; *n* = 7).

No statistically significant disturbances in the average time and distance needed to find the platform in all study groups were observed compared to the control group;however, it should be emphasized that FLU mice showed the most deviated time and distance parameters in relation to the other groups (Figure 4A,B). Moreover, the time spent in the W channel for FLU mice was significantly shorter compared to the INU group (25.80 ± 2.930 and 46.78 ± 4.821; * *p* < 0.05, *n* = 7; Figure 4C).

**Figure 5:**
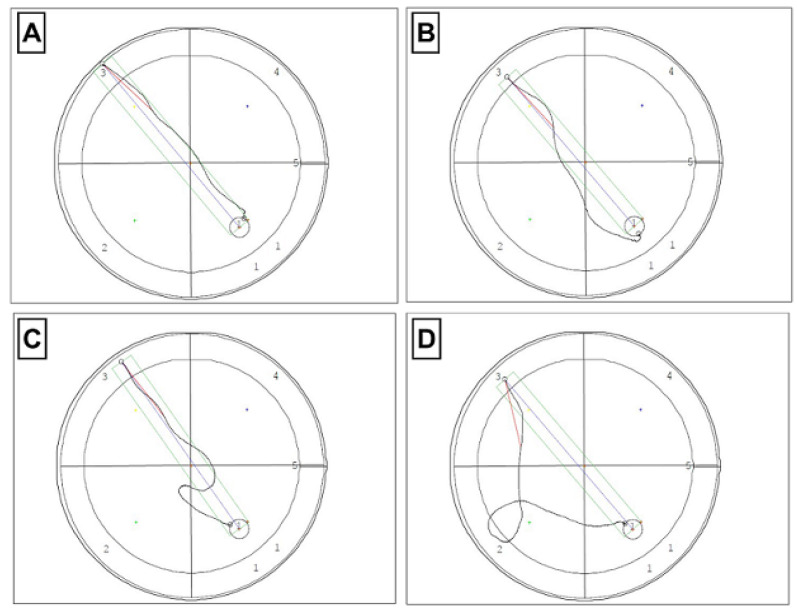
The recordings of the paths of directional flow in the W-channel for one selected animal from each study group. (**A**) control group; (**B**) TPB (Topinambur); (**C**) INU (Inulin); (**D**) FLU (Fluoxetine). The recording was made using a camera and a computer system with the Video Mot2 System software by TSE System.

The analysis of W-channel data recorded with the use of Video Mot2 System software made it possible to visualize the directional flow paths for individual groups. Figure 5 shows a record of the route taken in the W-channel for a selected animal from each study group starting from the 3rd quadrant.

### 3.3. The Impact of Long-Term Treatment with TPB, INU and FLU on the Neurogenesis in the SGZ and GCL in Mice

**Figure 6:**
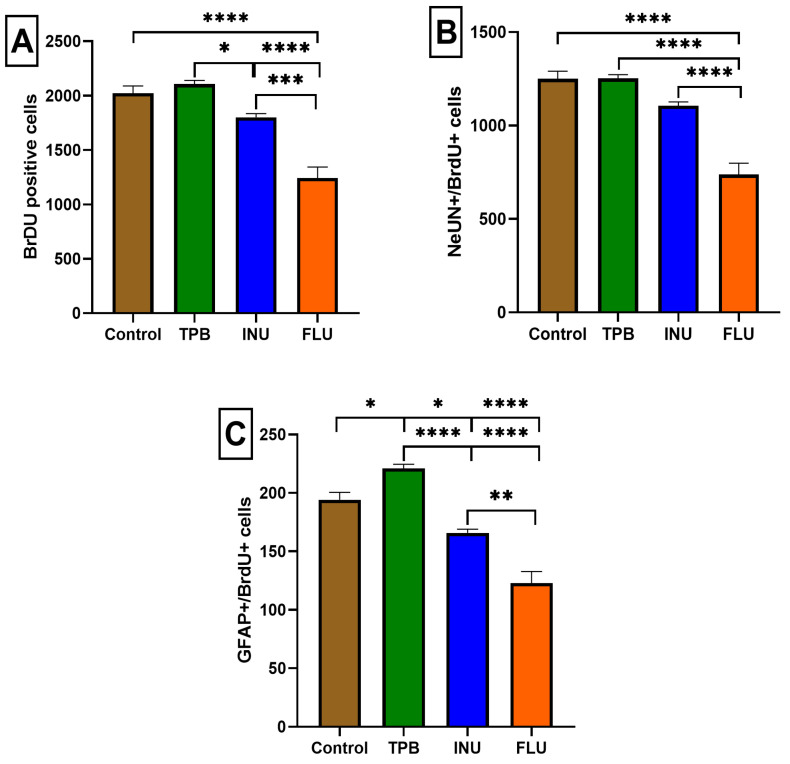
Quantitative analysis of the neurogenesis process after long-term treatment with TPB, INU, and FLU in mice. The effect of long-term treatment with TPB, INU, and FLU on the total newborn cells (**A**), newborn neurons (**B**), and newborn astrocytes (**C**) in the dentate subgranular zone of treated mice. The numbers of cells represent an estimate of the total number of positively labeled cells in the subgranular zone in both hemispheres. TPB—topinambur; INU—inulin; FLU—fluoxetine. Each bar represents the mean for five mice; error bars are S.E.M. (* *p* < 0.05,** *p* < 0.01, *** *p* < 0.001, **** *p* < 0.0001; *n* = 5).

To determine whether TPB, INU, and FLU administration may affect the number of progenitor cells in the SGZ and GCL, BrdU, (a thymidine analog that is incorporated into DNA during cell division) was used to label dividing cells. Quantification of BrdU-labelled cells showed that 2-week administration of FLU at a dose of 12 mg/kg statistically significantly reduced the number of BrdU-positive cells compared to the control group, TPB, and INU (1242 ± 102.2 vs. 2022 ± 66.24, 2106 ± 33.73, 1801 ± 33.79, respectively, *p* < 0.0001, *p* < 0.0001, *p* < 0.001, *n* = 5, Figure 6A). Interestingly, 10-week INU administration significantly decreased the total number of BrdU-positive cells compared to TPB (1801 ± 33.79 vs. 2106 ± 33.73, respectively, *p* < 0.05, *n* = 5; Figure 6A).

In order to assess possible changes in the proliferation, migration, and differentiation of newly formed cells labeled with the BrdU marker into neurons (NeuN) and astrocytes (GFAP), immunohistochemical staining of colocalization of BrdU/NeuN and BrdU/GFAP-positive cells was performed. The analysis of the obtained results showed that the number of cells labeled with BrdU/NeuN was significantly reduced in mice receiving FLU compared to the control group, TPB and INU (737.6 ± 60.63 vs. 1250 ± 40.95, 1252 ± 19.95, 1106 ± 20.72 respectively, *p* < 0.0001, *n* = 5, Figure 6B).

The labeling of a specific GFAP protein co-localizing with BrdU allowed visualization of the differences in the level of newly formed astrocytes in the hippocampus. A statistically significant reduction in the number of GFAP+ cells was observed in the FLU group compared to the control, TPB, and INU groups (122.8 ± 10.03 vs. 194.2 ± 6.351, 221.0 ± 3.479, 165 ± 3.169, respectively, *p* < 0.0001, *p* < 0.0001, *p* < 0.01, *n* = 5, Figure 6C). Interestingly, INU mice showed a significantly lower level of astrocytes compared to control and TPB mice (165 ± 3.169 vs. 194.2 ± 6.351, 221.0 ± 3.479, respectively, *p* < 0.05, *p* < 0.0001; *n* = 5, Figure 6C). Representative images of immunohistochemical changes for each of the study groups (control, TPB, INU, FLU) were included in the Supplementary File (Figures S1–S4).

### 3.4. Effect of Long-Term Administration of TPB, INU, and FLU on the Composition of the Intestinal Microbiota in Mice

**Figure 7:**
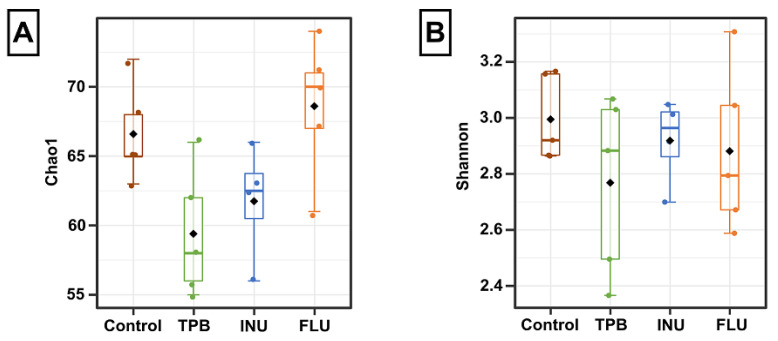
Alpha diversity indices calculated for different treatment groups. (**A**)—Chao1 index; (**B**)—Shannon index. The results were analyzed using the Kruskal–Wallis test. Data are presented as the mean ± SE.

To evaluate whether the gut microbiome was changed by TPB, INU, and FLU administration in mice, we conducted an in-depth analysis of 16S rRNA sequencing. The alpha diversity analysis revealed differences within the composition of the microbial community of each group (Figure 7A). Compared to the control group, the Chao1 index for INU and TPB groups decreased, whereas, for FLU mice, an increase was observed (H = 8.0849, *p* = 0.04). In turn, Shannon diversity indices were more similar and did not show significant differences.

**Figure 8:**
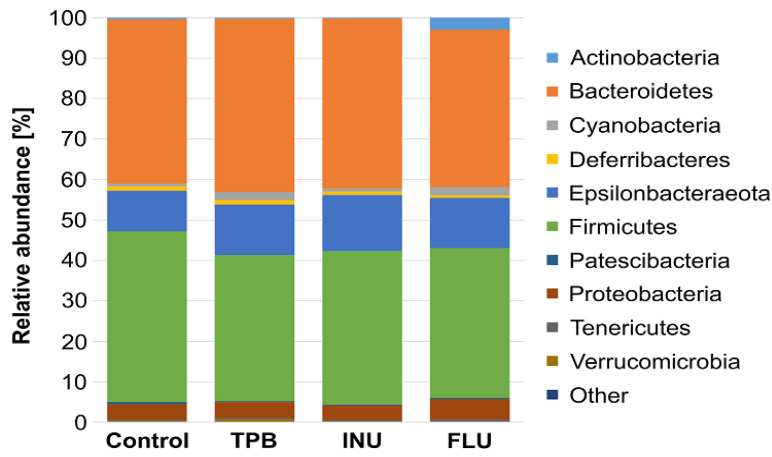
Relative abundance of bacterial phyla identified in tested groups of samples.

**Figure 9:**
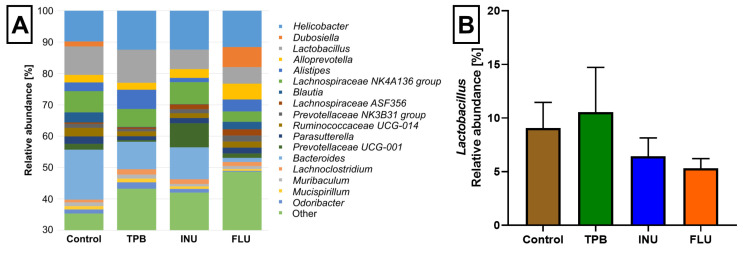
(**A**) Relative abundance of bacterial genera identified in at least two tested groups of samples with the abundance above 1%. (**B**) Relative abundance of *Lactobacillus*.

Across all tested samples, four phyla were the most abundant: *Bacteroidetes*, *Firmicutes*, *Epsilonbacteraeota* and *Proteobacteria* (Figure 8). Moreover, the abundance of *Actinobacteria* was higher in the FLU group than in other sample sets. At the genus level, seventeen identified genera were observed with anabundance higher than 1% in at least two tested groups (Figure 9). Their abundances differed among tested microbiota profiles (Supplementary Table S1). For example, *Dubosiella* was observed only in control and FLU groups, and *Blautia* was more abundant in those two groups than in INU and TPB (3.22% in control, 2.39% in FLU, 0.09% in INU, and 0.12% in TPB). In turn, the genus *Bacteroides* was observed in all but one profilewith a relative abundance above 8%. Only in the FLU profile was its abundance lower (1.30%). It was also observed that the abundance of *Lactobacillus* was the highest in the TPB group, whereas it was significantly reduced in the FLU group compared to the control group.

Results of ANOSIM analysis confirmed lower distances among intragroup samples than among intergroup samples (R = 0.342, *p* = 0.001).

**Figure 10:**
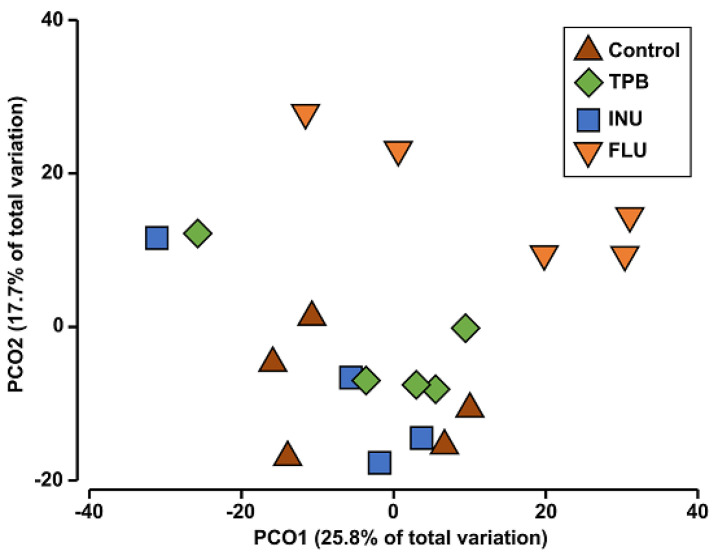
Principal–coordinate analysis (PCoA) showing a two–dimensional ordination of tested microbiota profiles grouped according to the treatment.

The PCoA analysis based on the abundances of all identified microbial taxa showed a high similarity of the tested microbiota profilegroups according to the treatment except FLU mice, where the subtle separation was observed (Figure 10). Moreover, results from pairwise PERMANOVA analysis supported also FLU mice separation (Table 1).

The results of the SIMPER analysis showed differences between the microbiome of the FLU groups and the other three profiles tested. Calculated overall average dissimilarities between profiles of compared groups were as follows: 48.65% for FLU-control, 50.95% for FLU-INU, and 49.40% for FLU-TPB. In turn, in all three comparisons (i.e., FLU-control, FLU-INU, and FLU-TPB), nine bacterial genera were identified as those shaping differences between groups’ microbiota (*Helicobacter*, *Bacteroides*, *Lactobacillus*, *Dubosiella*, *Blautia*, *Lachnospiraceae NK4A136* group, *Alloprevotella*, *Bifidobacterium*, and *Anaerostipes*, respectively). In the case of FLU-INU comparison, an additional four genera were linked with observed differences (*Alistipes*, *Prevotellaceae UCG-001*, *Lachnospiraceae UCG-001*, and *Prevotellaceae NK3B31*). In turn, differences between FLU and TPB groups were linked to the abundance of unidentified representatives of the *Gastranaerophilales* order and *Desulfovibrionaceae* family.

**Figure 11:**
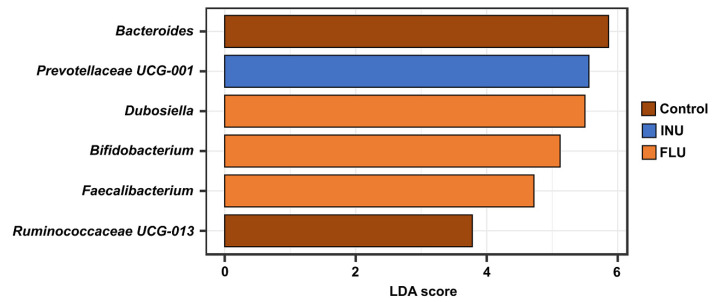
LEfSe analysis of the gut microbiota profiles grouped according to the treatment.

Based on the LEfSe analysis performed, six taxa of bacteria were identified to explain the difference in gut microbiota between the different treatment groups (Figure 11). The results showed that the biomarker taxa in the control group at the genus level were *Bacteroides* and *Ruminococcaceae UCG-013.* The biomarker genera in the FLU groups were *Dubosiella*, *Bifidobacterium*, and *Faecalibacterium*. In turn, for INU group *Prevotellaceae UCG-001* was identified as a marker genus. Only for the TPB group did LEfSe not determine the biomarker taxa.

Histogram of the LDA scores reveals the most differentially abundant taxa among tested groups.

## 4. Discussion

Over the past few years, there has been an increasing interest around the interactions between the gut microbiota and the effects of supplementation, including prebiotics as well as drugs in various therapeutic areas. In this study, we assessed thepotential impact of orally administered natural prebiotics—TPB and INU, and the antidepressant drug—FLU on learning and memory, neurogenesis, and the composition of the intestinal microbiota in healthy mice. The research results proved that the 10-week supplementation with TPB and INU stimulates the growth of probiotic bacteria, with no negative effect on the cognitive functions and the proliferation of neural stem cells in the tested animals. A 2-week administration of FLU confirmed its negative impact onbehavioral functions and neurogenesis. Moreover, we have also demonstrated an inhibitory effect of FLU on the growth of *Lactobacillus*.

Our research indicated no significant negative effect of TPB, INU, and FLU on the weight of the tested animals, which is consistent with the results obtained by Koch and coworkers [61] using supplementation with INU/FOS in C57BL/6 mice. Similarly, research by Petersen et al. [62] in BALB/c mice provided information that a 3-week supplementation of 10% of the diet with INU, FOS, XOS, GOS, apple pectin, polydextrose, or beta-glucan didn’t disturb the body weight of the tested animals. In turn, results from clinical trials showed, that FLU given chronically at a dose of 60 mg/day might have a modest effect on weight loss compared to a placebo in adults with overweight or obesity, although it may also induce many adverse events [63].

The MWM test was performed to evaluate the cognitive functions of healthy mice after INU, TPB, and FLU administration. The results of our experiment showed that mice receiving FLU covered a longer route and needed much more time to locate the platform than TPB, INU, and control mice, which indicated memory and learning impairments in the FLU group. In addition, the FLU mice spent definitely less time in the W-channel compared to the other groups, suggesting an impairment in spatial recognition. The effect of treatment with SSRIs such as FLU on behavior has been the subject of numerous studies in recent years. In vivo studies indicated that chronic therapy with FLU may have a very different effect on the behavior of treated rats or mice, depending on disease/dysfunction/health condition. The results we obtained are consistent with the studies of Majlesii and Naghdi [64] showing that FLU at doses of 8 and 16 mg/kg and citalopram at doses of 4 and 8 mg/kg significantly impaired the performance of the rats in the MWM test compared to the control animals. Interestingly, results obtained by Golub et al. [65] from the study on male juvenile rhesus monkeys indicated that 2 years of treatment with the drug impaired a sustained attention. Although response accuracy has not been affected, FLU monkeys had more missed trial initiations and choices during testing than control animals. On the contrary, FLU (5 mg/kg) treatment used in rat model of Alzheimer’s disease (AD) has been shown to increase learning and memory processes when compared with control AD rats. It should be emphasized, that FLU used in the treatment of neurological diseases responsible for cognitive dysfunctions has a beneficial effect, and therefore can improve the ability to learn and remember.

Taking into account the TPB and INU groups, the obtained results for all measured parameters were similar to the control mice, which confirms no impact of supplementation on learning and memory functions. Interestingly, the studies by Messaoudi et al. [66] have provided information that INU may significantly improve cognitive functions. They observed that a 14-day administration of a 5% or 10% mixture of INU and oligofructose inmale Wistar rats had a beneficial effect on the behavior of the tested animals. In turn, enriching the diet with INU enhanced the effect of the probiotic bacteria (*Enterococcus faecium*) and improved learning and memory in Sprague-Dawley rats [67]. Moreover, preliminary results from human studies conducted on young and middle-aged volunteers confirmed that the consumption of prebiotics such as FOS and INU, in doses of 5–10 g per day for 4–12 weeks, may be beneficial for brain function improvement (i.e., learning and working memory) and behavior (i.e., anxiety and mood) [68,69,70]. Taking into account the fact that TPB contains a significant amount of INU, the results of our research prove the beneficial effect of TPB oncognitive functions.

In the next part of the study, we evaluated, for the first time, the potent impact of TPB, INU, and FLU administration on the process of proliferation, migration, and differentiation ofthe neural stem cells in the mouse brain. The results of our study showed that a 10-week diet with TPB and INU did not affect the neurogenesis process in mice compared to the control group. At the moment there are no scientific reports concerning the effect of prebiotic administration on the course of the neurogenesis process. Interestingly a 2-week FLU administration significantly disturbed the process of neurogenesis. Similar to our current data, results from the study by Klomp et al. [71] showed an inhibitory effect of a 3-week administration of FLU (5 mg/kg) on the process of neurogenesis in rats. Moreover, in several other studies conducted on rodents, the lack of a stimulating effect of antidepressants such as FLU on the neurogenesis process was observed [72,73]. In turn, contrary results were presented by Marcussen et al. [74], where a 28-day treatment of FLU (10 mg/kg)was shown to stimulate neurogenesis in healthy rats. Similarly, the obtained results by Hovorka et al. [75] showed that a 14-day administration of FLU (4 mg/kg) induces the formation of new cells in growing rats. In addition, Hodes and colleagues [76] showed that 26-day treatment of female mice with FLU at doses of 5 and 10 mg/kg increased cell proliferation, whereas a low dose of FLU (2.5 mg/kg) did not affect the neurogenesis process in the tested animals [76].

As the final stage of the research, 16S rRNA sequencing was performed to assess changes in the gut microbiome of mice. We observed that the intestinal microbiota of mice receiving FLU was significantly different from the other study groups. The following bacterial taxa affecting differences in the gut microbiota were identified: for the FLU group *Dubosiella*, *Bifidobacterium*, and *Faecalibacterium*; for INU mice *Prevotellaceae UCG-001*, and for the control group *Bacteroides* and *Ruminococcaceae UCG-01*. Surprisingly, the biomarker taxa could not be determined for the TPB group. Additionally, four types of bacteria were most abundant in all tested groups: *Bacteroidetes*, *Firmicutes*, *Epsilonbacteraeota*,and *Proteobacteria*. Interestingly, the abundance of *Lactobacillus* was noticeably higher in the TPB group compared to the control group. Similar results were obtained in our previous initial screening study in male Albino Swiss mice [41], where 4-week supplementation with TPB (250 mg/kg) stimulated the growth of one of the most common probiotic bacteria, *Lactobacillus gasseri*, as well as *Enterobacteriaceae* (*Escherichia coli*, *Enterobacter asburiae*, *Kliebsiellaoxytoca*). The beneficial impact of TPB on the microbiota can be explained by the high content of INU in TPB tubers and as has been shown so far thatINU stimulates the growth of probiotic bacteria of the genus *Lactobacillus* and *Bifidobacterium* [77].

The results of our study are also in line with an earlier experiment by Samal et al. [43], evaluating the effect of a 12-week supplementation with TPB tuber meal (0, 2, 4, 6%) on the growth of probiotic bacteria in the intestines of rats. They showed that the enrichment of the diet in TPB increased the *Lactobacillus* spp. population and *Bifidobacterium* spp. in the cecum, colon, and anus.

Bioinformatic analysesfrom our experiment allowed us to study the effect of 2 weeks of FLU administration on the composition and number of the intestinal microbiota. One of the more important effects of FLU was the inhibition of the growth of *Lactobacillus*. Similar data, although from in vitro studies by Cussotto et al. [35], indicated that FLU at doses of 400 and 600 μg/mL completely stopped the growth of *L. rhamnosus*. In addition, at doses of 100, 400, and 600 μg/mL, it inhibited the multiplication of *Escherichia coli*. Further in vitro studies indicated that FLU and other SSRIs, that is, sertraline and paroxetine, exhibited antimicrobial activity against some Gram-positive bacteria, including *Staphylococcus* and *Enterococcus* [78,79]. Interestingly, Lyte et al. [36] noted a decrease in the number of *Lactobacilli* after 29-day treatment with FLU (20 mg/kg) in mice, although only some of the *Lactobacillus* strains were reduced, which may indicate a differential effect of FLU on different *Lactobacillus* strains. However, it should be mentioned that some of the results presented by other researchers differ from our reports. We observed a decrease in the number of *Lachnospiraceae NK4A136* in the FLU group compared to the control mice, whereas research by Lyte et al. [36] showed that several OTUs belonging to the family *Lachnospiraceae* (OTUs 32, 38, 86, 93) were significantly more numerous in FLU-treated mice.

Another bacterial taxon distinguishing the FLU group in our study was *Faecalibacterium*. It is well known that this bacterium is involved in the production of butyric acid, the main SCFA produced by the intestinal flora [80]. Moreover, in the studies involving patients with major depressive disorder (MDD), this bacterium was identified as a potential MDD biomarker [81]. Similarly, Zhou et al. [82] showed that both *Faecalibacterium* and *Butyricicoccus* may be important in diagnosing and treating patients suffering from postpartum depression.

Our findings revealed that the dominant type of bacteria in the mouse gut microbiota, *Bacteroides*, was most abundant in the control, INU, and TPB groups. The lowest amount was observed in the FLU group. Studies conducted so far have shown that *Bacteroides* metabolize polysaccharides and oligosaccharides, providing food and vitamins to the host and other intestinal bacteria. It is also known that these bacteria maintain a complex and generally beneficial relationship with the host when they remain in the intestine, but if they change the localization they can become pathogenic [83]

Analyzing the microbiome results obtained for the INU group, we observed an increase in the number of *Prevotellaceae UCG 001*. These results are in line with the study by Song et al. [84] where 4 weeks of INU (10 g/kg/day) supplementation increased the abundance of the family *Prevotellaceae* in C57BL/6J *ob*/*ob* mice, which are deficient in the leptin gene. In the same study, it was noted that the administration of INU also increased the amount of *Prevotellaceae UCG 001* in mice. *Prevotella*, as a succinate-producing bacterium, may participate in the decomposition of INU [85]. In addition, it has enzymes that are responsible for the degradation of cellulose and xylan [86]. On this basis, we can assume that enriching the diet with INU has a beneficial effect on the increase in the number of *Prevotella UCG-001*.

The analysis of the results obtained from our research, namely the effect of prebiotic supplementation on the microbiota, the BGM axis, and thus on cognitive functions and neurogenesis in mice, undoubtedly confirms the dependence of healthy brain function on proper homeostasis and gut-brain communication. Metabolites produced by the intestinal microbiota, such as SCFAs, amino acids, modified peptides, or oligosaccharides, may have an impact on the improvement of cognitive functions, which was indicated in animal models of neurodevelopmental and neurodegenerative diseases [87,88]. In turn, results from the study by Ogbonnay et al. [20] clearly proved the importance of microbiota for a proper neurogenesis. They indicated the altered levels of hippocampal neurogenesis in GF mice.

The results of our research showed that the bacterial taxa involved in the production of these secondary metabolites are primarily *Faecalibacterium* and *Bacteroides*. Research by Bravo et al. [11] showed that *Lactobacillus* regulates emotional behavior and central GABA receptor expression in mice via the vagus nerve. Moreover, Liang et al. [89] reported that *Lactobacillus helveticus* NS8 improved behavioral and cognitive impairments in rats. In this study, we observed a reduction in the abundance of *Lactobacillus* in fecal samples of the FLU group, which may be a potential contributor to cognitive impairment.

At the moment, our knowledge of the metabolic properties of many strains of bacteria is still limited. Therefore, it is extremely important to continue and implement advanced research in order to understand the significance of supplementation in the proper functioning of the microbiota and the BGM axis, especially in the context of exposure to various environmental factors that can induce dysbiosis.

## 5. Conclusions

In conclusion, the presented research showed that a long-term diet enriched with TPB or INU stimulates the growth of probiotic bacteria, does not affect the learning and memory process, and it does not disturb the proliferation of neural stem cells in the tested animals. Based on this data we can assume that both TPB and INU seem to be safe for the proper course of neurogenesis. On the contrary, 2-week administration of FLU confirmed an inhibitory impact on *Lactobacillus* growth and negatively affected behavioral function and neurogenesis in healthy animals.

The above studies suggest that the natural prebiotics TPB and INU may have the potential to become natural supplements that enrich the diversity of intestinal microbiota while having a beneficial effect on the BGM axis, cognitive functions, and neurogenesis, which is certainly worth further advanced preclinical research.

## Figures and Tables

**Table 1:** Results of pairwise PERMANOVA.

Compared Groups	Pseudo-F	*p*
control—FLU	1.9887	0.007 *
control—INU	1.2850	0.103
control—TPB	1.2337	0.152
FLU—INU	1.7685	0.010 *
FLU—TPB	1.5593	0.007 *
INU—TPB	1.1114	0.260

Significant differences between compared groups are marked with asterisks (* *p* < 0.05).

## Data Availability

The data supporting reported results can be found in the laboratorydatabases of Institute of Rural Health.

## Supplementary Materials

The following supporting information can be downloaded at: https://www.mdpi.com/article/10.3390/cimb45030168/s1, Table S1: The total composition of the intestinal microbiota in mice, Representative images of immunohistochemical changes for Control (Figure S1), FLU (Figure S2), INU (Figure S3), TPB (Figure S4).
