# Supplementary figures and images for: The Effect of a Diet Enriched with Jerusalem artichoke, Inulin, and Fluoxetine on Cognitive Functions, Neurogenesis, and the Composition of the Intestinal Microbiota in Mice
Source: Curr Issues Mol Biol. 2023 Mar 21;45(3):2561–79. doi: 10.3390/cimb45030168 (PMC10047150; doi:10.3390/cimb45030168)

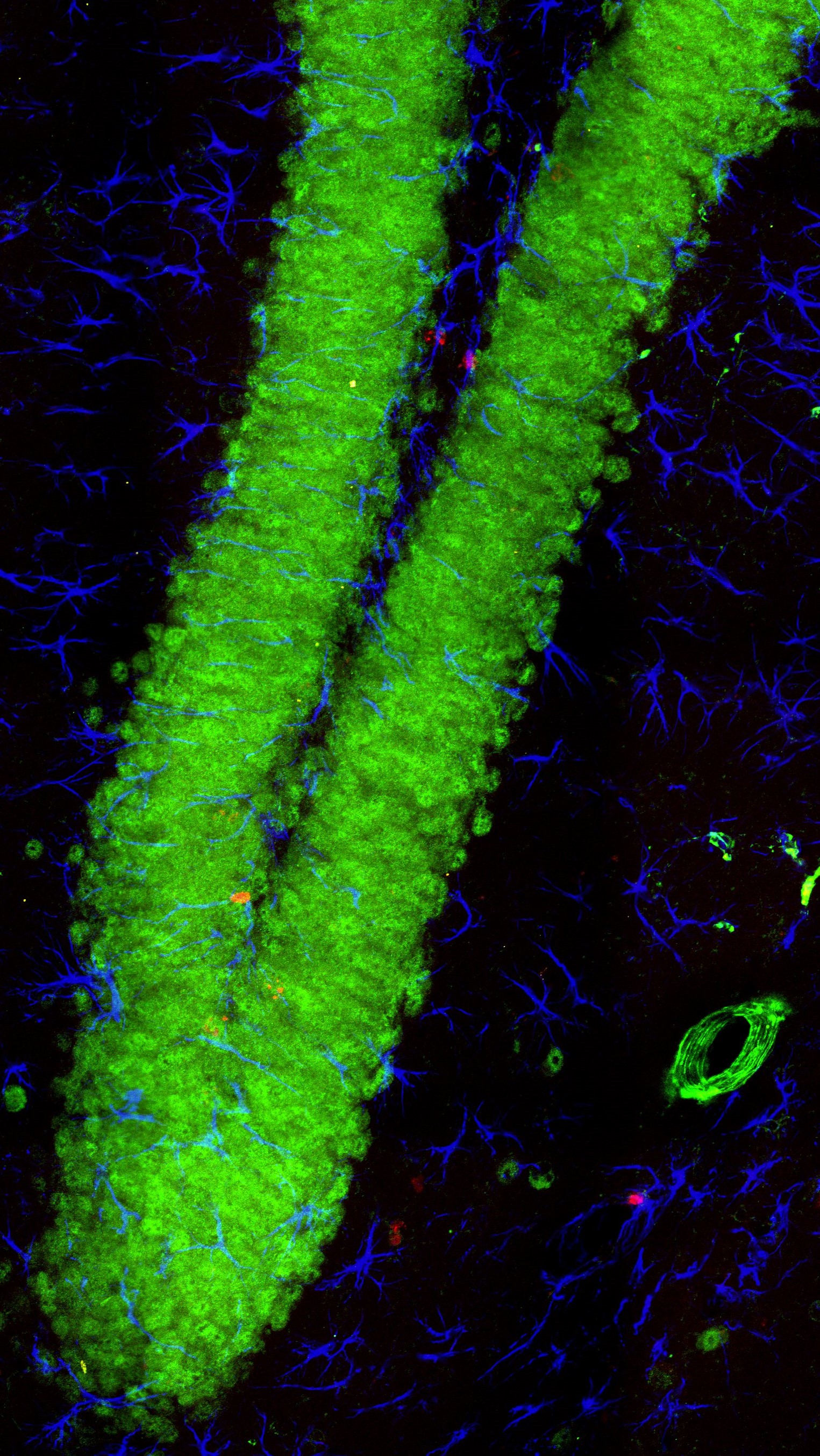

Supplement: Supplementary file 1 [file cimb-45-00168-s001.zip › Figure S1 Control.jpg]

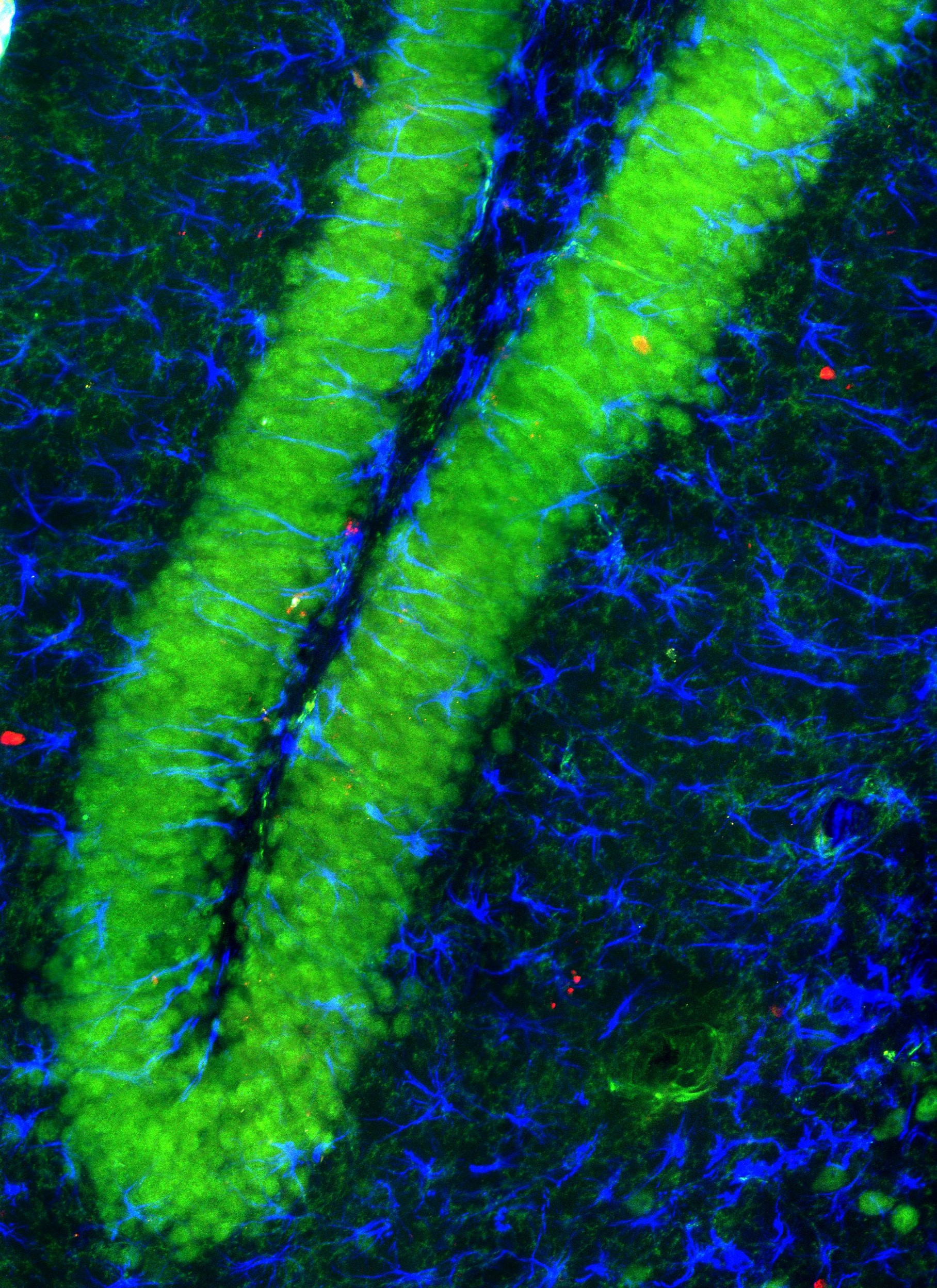

Supplement: Supplementary file 1 [file cimb-45-00168-s001.zip › Figure S2 FLU.jpg]

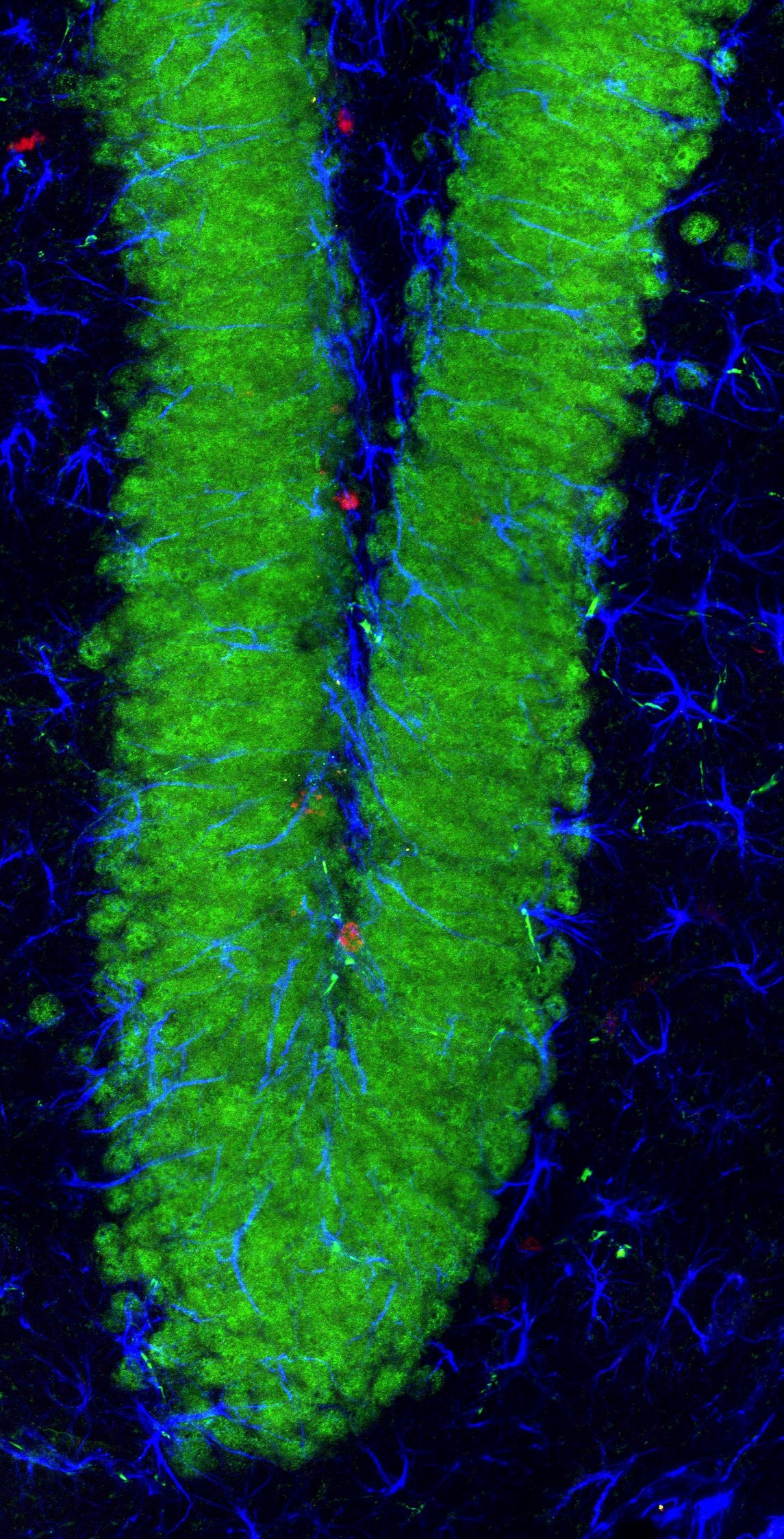

Supplement: Supplementary file 1 [file cimb-45-00168-s001.zip › Figure S3 INU.jpg]

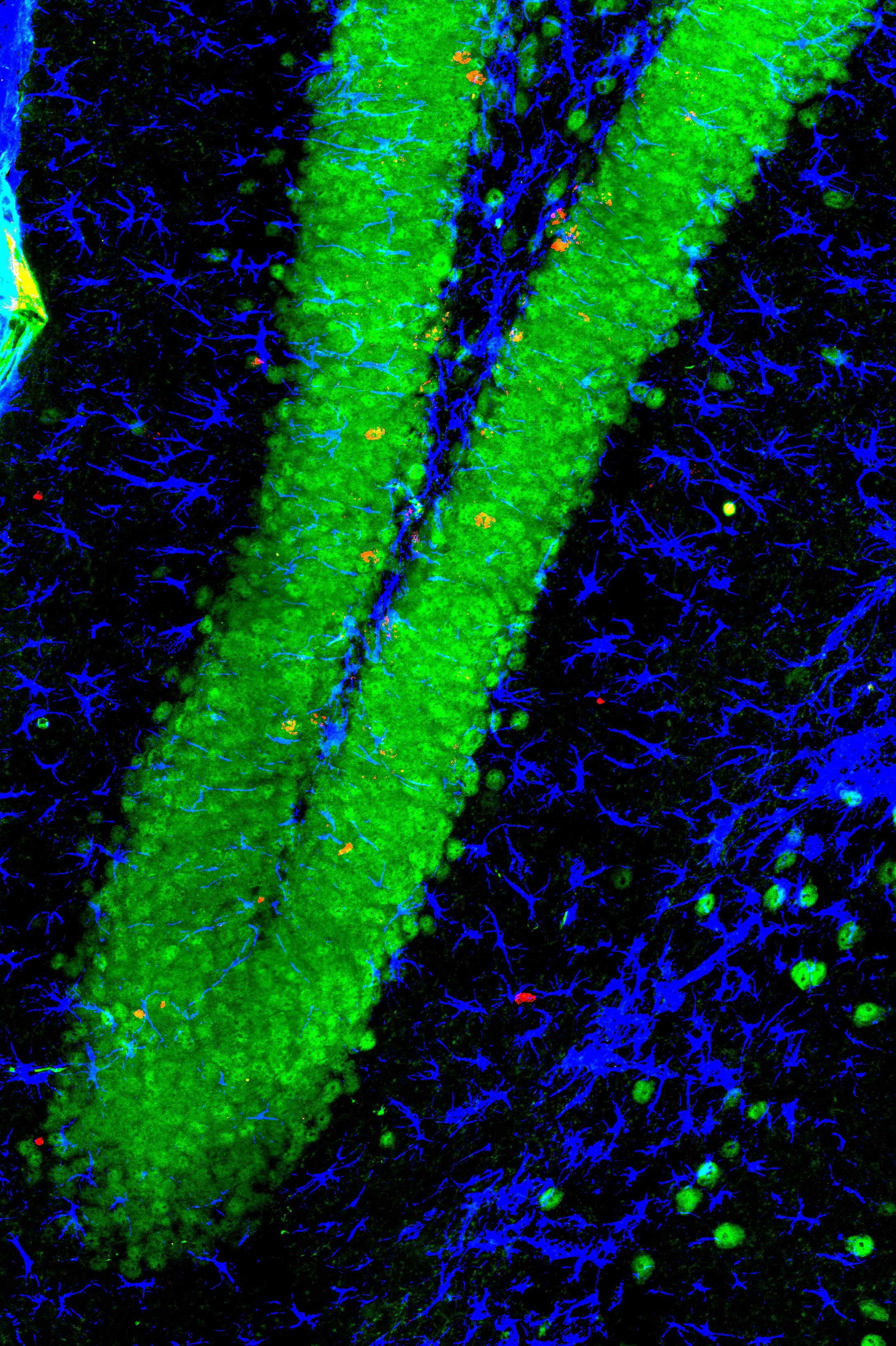

Supplement: Supplementary file 1 [file cimb-45-00168-s001.zip › Figure S4 TPB.jpg]
